# Home visits to improve breast health knowledge and screening practices in a less privileged area in Jordan

**DOI:** 10.1186/1471-2458-14-428

**Published:** 2014-05-06

**Authors:** Hana Taha, Lennarth Nyström, Raeda Al-Qutob, Vanja Berggren, Hamideh Esmaily, Rolf Wahlström

**Affiliations:** 1 Department of Public Health Sciences, Global Health (IHCAR), Karolinska Institute, Stockholm, Sweden; 2 Jordan Breast Cancer Program, Amman, Jordan; 3 King Hussein Cancer Foundation, Amman, Jordan; 4 Division of Epidemiology and Global Health, Department of Public Health and Clinical Medicine, Umeå University, Umeå, Sweden; 5 Women and Child Health Division, Department of Family and Community Medicine, University of Jordan, Amman, Jordan; 6 Faculty of Health Sciences, Lund University, Lund, Sweden; 7 Family Medicine and Preventive Medicine, Department of Public Health and Care Sciences, Uppsala University, Uppsala, Sweden

## Abstract

**Background:**

Breast cancer is the most common cancer afflicting women in Jordan. This study aimed to assess the effects of an educational intervention through home visits, including offering free mammography screening vouchers, on changing women’s breast health knowledge and screening practices for early detection of breast cancer in a less privileged area in Jordan.

**Methods:**

Two thousand four hundred breast health awareness home visits were conducted and 2363 women aged 20-79 years (median: 41) answered a pre-test interview-administrated questionnaire to assess their breast health knowledge and practices at the baseline. After a home-based educational session, 625 women aged 40 years or older were referred to free mammography screening. Five hundred and ninety six homes were revisited six months later and out of these 593 women participated in a post-test. The women’s retained breast health knowledge, the changes in their reported breast health practices and their usage of the free mammography voucher, were assessed.

**Results:**

The mean knowledge score increased significantly (p < 0.001) from 11.4 in the pre-test to 15.7 in the post-test (maximum score: 16). At the six month follow-up the post-test showed significant (p < 0.001) improvement in women’s perceived breast self-examination (BSE) knowledge, reported BSE practice and mammography screening. Out of 625 women that received a voucher for free mammography screening 73% attended the mammography unit, while only two women without a voucher went for mammography screening at the assigned unit. Women who received a follow-up visit were more likely to use the free mammography voucher compared to those who were not followed-up (83% vs. 67%; p < 0.001).

**Conclusions:**

Home visits by local community outreach workers that incorporated education about breast cancer and breast health in addition to offering free mammography screening vouchers were effective in improving women’s breast health knowledge and practices in a less privileged area in Jordan.

## Background

Breast cancer is the leading cause of cancer death and the most common female cancer worldwide, accounting for 23% (1.4 million) of the total new cancer cases and 14% (458,400) of the total cancer deaths [1]. It is estimated that 1.7 million women will be diagnosed with breast cancer in year 2020, and the majority of these cases will be in low- and middle income countries (LMIC) [2]. Early detection of breast cancer is essential for LMIC, because breast cancer at early stages has a better prognosis with more cost-effective treatment [3].

Breast cancer is the most common cancer afflicting women in Jordan, comprising nearly 20% of all incident cancer cases and 37% of all female cancers [4]. In 2009, 43% of the cases diagnosed were in women aged 20-49 years and 36% in those aged 50 years and more; 3.0% of the cases were in stage 0 (in situ), 26% in stage I, 30% in stage II, 23% in stage III, and 10% in stage IV [4]. During the period from 1997 to 2002 the five-year survival rate of breast cancer in Jordan was 83% for stage I, 72% for stage II, 59% for stage III, and 35% for stage IV cancers [5]. Based on this data it was suggested that creating breast health awareness in addition to conducting targeted mammography screening interventions for women aged 40 years and above might lead to earlier detection of breast cancer and thus to higher survival rates [5].

The Jordan Breast Cancer Program (JBCP) was established in 2007 as a national initiative led by the King Hussein Cancer Foundation (KHCF) to improve public awareness about breast cancer and the benefits of early detection and to ensure the provision of quality screening services in Jordan [6]. The JBCP orchestrates the efforts of all the stakeholders in the different sectors to enhance women’s breast health awareness and their access to quality screening services [6]. The national guidelines for early detection of breast cancer promote breast health awareness, including monthly practice of breast self-examination (BSE) starting from the age of 20 years [6]. Clinical breast examination (CBE) is recommended once every 1-3 years in the age group 20-39 years and annually in women aged 40 years and older. Mammography is recommended once every 1-2 years for all women starting from age 40 years.

Women in Jordan, especially those who live in less privileged areas, might still face barriers that constrain them from adopting screening practices [6]. These barriers could include prioritizing the needs of the children and family above their own health, having fears about breast cancer and feeling safe and not at risk of the disease. To enhance women’s access to culturally appropriate information about breast cancer and to overcome possible barriers to their breast health practices, the JBCP in collaboration with the American Near East Refugees Aid (ANERA) and the Jordanian Women’s Union (JWU) conducted a home visits intervention to women in a Palestinian refugees’ camp in Jordan. The intervention included a home-based educational session and offered free mammography screening vouchers. This study aimed to assess the effect of this outreach home visits intervention on changing women’s breast health knowledge and screening practices for early detection of breast cancer.

## Methods

### Study setting

Jordan hosts approximately two million registered Palestinian refugees and has ten official camps that house approximately 346 000 registered refugees [7]. This study was conducted in the second largest Palestinian refugees’ camp in Jordan [8]. The camp was set up in 1955 on an area of 488 000 square meters south-east of Amman. The refugees were initially accommodated in 1400 shelters, but over the years the camp has grown into an urban neighbourhood. Currently, there are more than 51 500 registered refugees living in the camp. The United Nations Relief and Works Agency for Palestine Refugees in the Near East (UNRWA) operates 13 schools, one community-based rehabilitation centre, one women’s program centre and one health centre in the camp. The health centre delivers primary health services including CBE. However, UNRWA does not offer or provide mammography services to beneficiaries. The circumstances in which UNRWA operates are becoming more challenging with the increased burden of non-communicable diseases among the Palestinian refugees due to aging. Other socio-demographic determinants that could negatively influence the health status of these refugees include crowdedness, early marriage and divorce, poverty, unemployment, lack of social security, and lack of secondary and tertiary health care insurance [9].

### Study design

This home visits intervention incorporated two components: a culturally appropriate home-based breast health educational session; and referral of women aged 40 years or older, who met the inclusion criteria, to a free-of-charge mammography screening at a nearby mammography unit.

**Figure 1:**
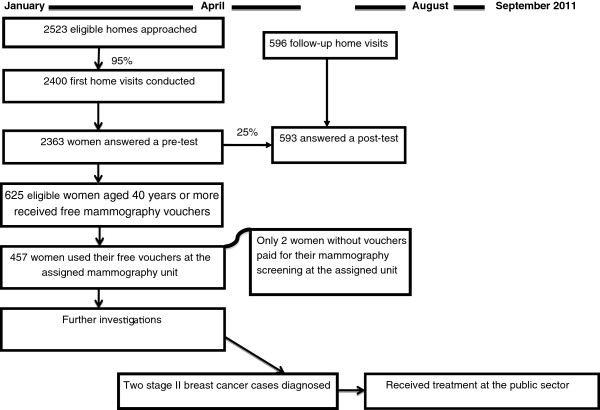
Flow chart of the intervention process.

The effects of the intervention were evaluated using a before and after study design that included: assessment of women’s breast health knowledge and breast cancer screening practices at the baseline (pre-test) and a post-test of women’s retained knowledge and their current screening practices at follow up visits six months after the first visit. Figure 1 shows a flow chart of the intervention process.

### Training of outreach workers

In December 2010 the JWU trained 22 female local community outreach workers (LCOWs) to conduct home visits to create awareness of breast cancer and breast health. The JWU had previous experience in conducting health awareness home visits in the targeted camp and had a network of trained LCOWs. The LCOWs were 25-35 years old, had at least high school education, and had previous experience of community outreach work in the camp. A JBCP certified female trainer conducted a three-days training workshop for the LCOWs. The first day of the training included: information about breast cancer statistics; risk factors; signs and symptoms; and national guidelines for breast cancer early detection examinations, including practical training on BSE. In the second day, the LCOWs were trained on communication and community health promotion skills. They were also taught how to address myths and socio-cultural barriers to women’s breast health practices. In the third day, the participants had practical sessions on how to use a specially designed culturally appropriate educational kit, and how to collect data using the study questionnaires.

Based on the trainer’s evaluation of their practical skills, six LCOWs were selected to conduct the home visits and another six were selected for assistance in the field. The remaining trained LCOWs were standby for any needs during the implementation of the study. The training was attended by two female data entry specialists from JWU and three female field supervisors from JBCP, ANERA and JWU. All the trained LCOWs were prepared to serve as grassroots leaders to enhance the sustainability of breast health awareness in the camp.

### Conducting home visits

Between 1 January and 30 April 2011, 2400 breast health awareness home visits were conducted by the trained LCOWs in the camp. This 2400 first home visits sample size was pre-set at the baseline because of funding limitations. The eligibility criterion for conducting a home visit was: a home within the camp that had at least one female aged 20 years or more. The camp was divided into six functional areas, and the homes were selected non-randomly in each of the six areas. The weekly home visits schedule of each of the six LCOW was set based on previous outreach experience of the JWU, and was discussed and approved by the field supervisors from JBCP and ANERA. To reach the targeted 2400 home visits, an additional 123 homes (4.9%) had to be visited, in order to replace those that could not be included for the following reasons: did not allow the LCOW to enter (n = 76, 3%); the female head of the household was busy (n = 33, 1.3%); or the house was closed (n = 14, 0.6%).

At the beginning of each home visit the LCOW interviewed the female head of the household and collected baseline data about her socio-demographic characteristics and her breast health knowledge and practices. The pre-test data was collected for one woman per home visit. In total, 2363 women aged 20-79 years (median: 41) answered the pre-test (response rate: 98%). In some visits the daughter or the daughter in law attended the session and was interviewed instead of the head of the household if she refused to participate. After that the LCOW used the educational kit to educate the women on breast cancer and breast health. In total 7759 women (median = 3) attended these home-based educational sessions.

### Educational intervention

A specially designed culturally appropriate educational kit was developed to be used by the trained LCOWs during their home visits. Each kit consisted of a flip cards presentation, visual aids, pre- and post-tests, referral vouchers to a free of charge mammography, and a breast model for demonstration on how to perform BSE. Each home visit lasted 70-90 minutes; 25-30 minutes for the interview and 45-60 minutes for the educational session. The educational session covered the following topics: what is breast cancer, breast cancer statistics in Jordan, breast cancer risk factors, signs and symptoms, benefits of early detection, how breast cancer is diagnosed, BSE training, CBE and mammography, breast health national guidelines, breast cancer treatment options and patient support groups in Jordan.

### Referral to free mammography screening

The LCOWs had 625 free mammography vouchers for an assigned mammography unit to offer to women who met the following eligibility criteria: 40 years or older; never had a mammography before or not during the last year; had at least one breast cancer risk factor; had no health insurance; or had an annual family income of less than 5475 USD annually, which was Jordan’s National Average Absolute Family Poverty Line for food and non-food in year 2008 for the average family size of 5.7 members [9]. The list of risk factors that made the woman eligible for the free mammography voucher was adapted from the Susan G. Komen’s breast cancer risk factors list [10] and included family history of breast, ovarian or prostate cancer (first degree relative); giving birth to the first child after the age of 35 years (or had not given birth); having a personal history of breast cancer or undergoing breast biopsies in the past; late onset of menopause after the age of 55 years; starting menstruation before the age of 12 years; previous radiation therapy; having taken hormonal replacement therapy after menopause; having used contraceptives within the last ten years; did not breastfeed her children; obesity based on having a body mass index of 30 or higher; lack of exercise or physical activity; eating fatty food and smoking. The number of 625 free mammography vouchers was pre-set at the baseline because of funding limitations.

Eighteen of the eligible women (3%), who were offered the free voucher refused to take it. When that happened the voucher was offered at another home visit to another eligible woman. Each referred woman was given an appointment to go for mammography screening within one month of the date of the home visit. All the referred women had the flexibility to re-schedule their appointments within a one-month time frame. The selected mammography unit was located nearby the camp, which made it accessible to the women. The unit met local and international standards with regard to comfort, privacy and encouraging atmosphere. It was staffed with an experienced radiologist and technician, who were both trained by JBCP. In addition, JBCP hired a consultant radiologist to perform double reading of the mammograms to ensure the quality of the screening.

### Follow-up visit

The LCOWs scheduled follow-up visits after six months (July 2011) for the 600 women in the homes that were first visited in January 2011 in order to assess their retained knowledge and changes in breast health practices during the past six months. The sample size of 600 follow-up home visits was pre-set at the baseline because of funding limitations. The LCOWs successfully conducted 596 follow-up visits and collected post-test data from 593 women aged 20-76 years (median: 42) (participation rate: 99%).

### Measure instruments

Pre- and post-test questionnaires were developed by the research team. The pre-test questionnaire included four sections: socio-demographic characteristics; breast cancer risk factors; ever had previous BSE, CBE and mammography screening; breast health knowledge based on 16 true or false statements; and participation in any previous lecture about breast cancer. The post-test questionnaire included three sections: breast health knowledge; BSE, CBE and mammography screening during the previous six months; and barriers to BSE, CBE and mammography screening. The knowledge section was the same in the pre-test and in the post-test and included 16 knowledge statements to be answered by true or false. In the practices section, the woman was asked about mammography screening, if she had had a CBE and if she had practised BSE during the past six months after the first visit. The questionnaires were validated and adjusted based on a previous educational intervention conducted by the research team [6], and on a previous home visit project conducted in a similar underprivileged area in Amman.

### Ethical issues

This outreach home visits intervention was approved by the Palestinian refugees’ authorities in Jordan and by Jordan’s Ministry of Social affairs. The ethical clearance for this study was issued from the Jordan Ministry of Health Research Ethics Committee. The confidentiality of the data and the autonomy of the participants were ensured. They were informed of the purpose of the study, the voluntary nature of their participation and their right to access findings. They were also informed about the possible benefits and harms of the intervention before giving their consent. Any sharing of the data within the research team included only numeric codes so that no individual participant could be traced. All the participants gave a verbal consent to the LCOW in the presence of a field assistant.

### Data analysis

The answers to the 16 breast health knowledge questions were coded as correct or incorrect. Each correct answer was given the weight of one point and the maximum score was 16 points. Paired t-test was used to assess whether there was any improvement in women’s breast health knowledge at six months’ follow-up. The effects of the educational intervention on women’s screening practices including BSE, CBE and mammography were determined using the chi-square test. The effect of receiving a free mammography voucher on the actual mammography practice was also assessed using chi-square and Fisher’s exact test. The data analysis was carried out using SPSS. The level of statistical significance was set at p < 0.05.

## Results

In this intervention, 55% of the women who received the first visit were aged 40 years or more; 81% had low family income; 74% had no health insurance; 96% were housewives; 80% were married; 8.7% were illiterate; 44% had primary or secondary education; 10% of the women’s husbands were unemployed while 54% of them worked as handymen or drivers (Table 1). Women who were not revisited had similar characteristics as those who received the follow-up visit except for small differences with regard to age groups, woman’s occupation and attending a previous lecture about breast cancer. In addition, the proportion of women with a low knowledge score in the pre-test was higher among the women who were revisited than among women not revisited (45% vs. 37%; p = 0.001).

**Table 1:** Basic characteristics of 2363 participating women and difference between revisited and not revisited women

		**All women**		**Not revisited**		**Revisited**		P-value*
**Characteristic**	**Category**	**n=2363**	**%**	**n=1770**	**%**	**n=593**	**%**	
Age group	20-29	474	20	365	21	109	18	0.033
	30-39	598	25	468	27	130	22	
	40-49	686	29	491	28	195	33	
	50-59	377	16	272	15	105	18	
	60-79	227	9.6	173	9.8	54	9.1	
Marital status	Single	127	5.4	98	5.5	29	4.9	0.32
	Married	1903	80	1435	81	468	79	
	Divorced	79	3.3	54	3.1	25	4.2	
	Widow	254	11	183	10	71	12	
Family income (JNAAFPL)**	< JNAAFPL	1913	81	1437	81	476	80	0.66
	≥ JNAAFPL	448	19	332	19	116	20	
Health insurance	No	1753	74	1324	75	429	72	0.24
	Yes	610	26	446	25	164	28	
Women Education	Illiterate	206	8.7	161	9.1	45	7.6	0.51
	Elementary	369	16	276	16	93	16	
	Secondary	665	28	488	28	177	30	
	High school	823	35	612	35	211	36	
	College and higher	300	13	233	13	67	11	
Husband’s education	illiterate	81	3.4	60	3.4	21	3.5	
	Elementary	363	15	267	15	96	16	0.57
	Secondary	628	27	486	27	142	24	
	High school	497	21	366	21	131	22	
	College and higher	794	34	591	33	203	34	
Women occupation	Housewife	2271	96	1709	97	562	95	0.040
	Other	87	3.7	57	3.2	30	5.1	
Husband’s occupation	Retired	113	5.9	87	6.1	26	5.5	0.35
	Unemployed	185	9.7	150	10.5	35	7.4	
	Handyman, driver	1024	54	758	53	266	56	
	Employee	382	20	286	20	96	20	
	Business owner	200	10	150	10	50	11	
Previous lecture	Yes	560	24	398	22	162	27	0.011
	No	1801	76	1370	77	431	73	
Pre-test knowledge	Scored 0-11	930	39	663	37	267	45	0.001
	Scored 12-16	1433	61	1107	63	326	55	

*Difference between women revisited and not revisited.

**JNAAFPL = Jordan National Average Absolute Family Poverty Line (food and non-food poverty) in 2008 amounted to 5475 USD annually for the average family size of 5.7 members. [http://www.dos.gov.jo/dos_home_a/main/Analasis_Reports/poverty_rep/poverty_rep_2008.pdf].

### Knowledge scores

The median percentage of correct answers on the 16 knowledge questions at the pre-test was 77% (Range: 37%-95%) for the 1770 women who did not receive a follow up visit and 75% (Range: 30%-97%) for the 593 women who later received a follow up visit. In the post-test the median percentage of correct answers was 99% (Range: 93%-100%). Among the revisited the mean knowledge score increased significantly (p < 0.001) from 11.4 in the pre-test to 15.7 in the post-test and the percentage of correct answers increased significantly for 14 out of 16 knowledge statements (p < 0.001) (Table 2).

**Table 2:** Percentage (%) of correct answers of the true and false knowledge statements in the pre- and post-tests

**Knowledge statement**	**Pre-test**		**Post-test**
	**Not revisited (n = 1770)**	**Revisited (n = 593)**	**Revisited (n = 593)**
***True (n = 11)***			
Breast cancer can be cured	74	66	99*
The probability of the woman getting breast cancer increases at the age of 40	69	61	99*
Breast cancer cure rate depends on its stage at detection	81	76	99*
Breast feeding protects the woman from breast cancer	78	79	98*
There is an association between obesity and breast cancer	37	30	93*
When a woman feels any abnormal changes in her breasts she should go to see her doctor	95	97*	100**
Practicing a monthly BSE helps the woman to notice any abnormal changes in the breast	92	92*	100**
A woman is advised to practice a monthly SBE starting from age 20	74	70	99*
During breast self-examination you need to examine the underarm	79	73	99*
All women are advised to seek clinical breast examination starting from age of 20	67	60	99*
A woman is advised to do a mammogram once every one or two years starting from the age 40	86	86	99*
***False (n = 5)***			
All breast lumps are cancer	83	79	98*
All nipples secretions are normal regardless of the colour	77	77	99*
Breast cancer is always associated with pain	56	60	94*
Getting breast cancer means mastectomy	51	55	93*
Early detection examinations are recommended only for married women	87	79	98*

*The percentage of correct answers increased significantly between the pre-test and the post-test for 14 statements (p < 0.001). **These two statements had high percentage of correct answers in both the pre- and the post-tests.

### Breast health practices

In total, about one fourth of the women had previously attended a lecture on breast health, had ever performed BSE, or had ever done CBE (Table 3). Less than half of the women (47%) perceived they had BSE knowledge, and less than one out of ten (7.9%) ever participated in mammography screening (among women aged 40 years and older). Breast health knowledge and practice was significantly higher among women with a knowledge score between 12 and 16 as compared with women with a knowledge score of eleven or lower (Table 3). Previous mammography screening was significantly associated with being aged 40 years or older, having an annual family income above 5 475 USD and having health insurance (p < 0.001).

**Table 3:** Pre-test breast health knowledge scores in relation to woman’s perceived breast self-examination (BSE) knowledge and breast health practices at the baseline

	**Knowledge score**				**Total**		P-value*
	**0-11 (n = 930)**		**12-16 (n = 1433)**				
	**n**	**%**	**n**	**%**	**n**	**%**	
Attended a previous lecture	164	18	164	18	560	24	<0.001
Ever had mammography screening *(Aged 40 years or more)*	30	5.4	72	9.8	102	7.9	0.004
Perceived to have BSE knowledge	326	35	795	55	1121	47	<0.001
Ever did BSE	141	16	433	31	574	25	<0.001
Ever had CBE	147	16	392	27	539	23	<0.001

*Difference between lower and higher breast health knowledge score levels.

At the first visit there was no difference in percentage women that perceived they had BSE knowledge, ever did BSE and ever had CBE between women not revisited and women revisited (Table 4). At six month follow-up percentage women that perceived they had BSE knowledge, practised BSE monthly had increased.

**Table 4:** Percentage reported perceived breast self-examination (BSE) knowledge and practices of BSE and clinical breast examination (CBE) in the pre and post- tests

**Reported knowledge and practices at the baseline**	**Pre-test**			**Reported knowledge and practices in the last six months**	**Post-test**	P-value*
	**All women (n = 2363) %**	**Not revisited (n = 1770) %**	**Revisited (n = 593) %**		**Revisited (n = 593) %**	
Perceived BSE knowledge	47	46	51	Perceived BSE knowledge	99	<0.001
Ever did BSE	25	24	27	Did monthly BSE	96	<0.001
Ever had CBE	23	22	26	Had CBE	29	0.22

*Difference between the pre-test and post-test for women revisited.

Receiving a free mammography voucher increased the likelihood of women’s mammography screening. Out of the 625 referred women 73% used their free vouchers and had their mammography done in the assigned unit. Out of 563 women who were aged 40 years or older and who never participated in mammography screening, but could not be offered a free voucher due to the limited resources two had mammography screening in the assigned unit and paid for it and 13 reported in the post-test that they had mammography in other mammography units.

Higher usage of the free mammography voucher was seen among the 246 women, who received a follow-up visit (203/246 = 83%) compared to those 379 women, who were not revisited (254/379= 67%) (p < 0.001). There was also higher usage of the free vouchers among women who reported in the pre-test that they attended a previous lecture about breast health, had a perceived BSE knowledge and practice and ever had a CBE (Table 5). Women who practiced CBE during the six months that followed the first visit were also more likely to use their free mammography screening vouchers (Table 5). However, there were no significant associations between the women’s use of the free voucher for screening, and their age, marital status, family income, education level, occupation, husband’s education, husband’s occupation.

**Table 5:** Use of the free vouchers in relation to women’s knowledge and reported practices

***At pre-test (n = 625):***	**Used the free mammography voucher**		p-value*
	**Yes**	**No**	
	**(n = 457)**	**(n = 168)**	
Ever attended a lecture	28%	14%	0.001
Perceived BSE knowledge	49%	32%	0.001
Ever did BSE	26%	17%	0.014
Ever had CBE	27%	13%	0.001
***At post-test (n = 246):***	**n = 203**	**n = 43**	
Had CBE	35%	12%	0.002

*Difference between women who used and those who did not use the voucher.

Reported barriers in the post-test for women’s BSE, CBE and mammography screening practices are shown in Table 6. Being busy and having other priorities was the most frequently reported barrier, in particular for CBE, while not feeling at risk was as important as a barrier to perform a mammogram. A few women mentioned being afraid of possible harms from the X-ray, not being encouraged by their families, not getting approval from their husband, and logistical obstacles at the health facility as barriers to attending mammography screening. There were also some women who refrained from both mammogram and CBE, as they placed their destiny in God’s hand. Less frequently mentioned barriers are listed in Table 6.

**Table 6:** Revisited women’s reported barriers to breast health practices in the post-test

Reported barriers*	**Mammogram n (%)**	**BSE n (%)**	**CBE n (%)**
***Revisited n = 593***			
I still don’t know how to do BSE	0	2 (0.34)	0
Busy and have other priorities	48 (8.1)	7 (1.2)	165 (28)
I do not feel that I am at risk of breast cancer	44 (7.4)	4 (0.67)	43 (7.3)
Human destiny is in God’s hand	24 (4.0)	5 (0.84)	18 (3.0)
I am afraid that x ray is harmful	12 (2.0)	0	0
I do not get any encouragement from my family	10 (1.7)	0	0
Because of logistical obstacles at the hospital	10 (1.7)	0	0
Busy with other illnesses	7 (1.2)	7 (1.2)	7 (1.2)
My husband did not approve my going for the test	6 (1.0)	0	3 (0.51)
I do not have anyone to accompany me	5 (0.84)	0	5 (0.84)
I am afraid from the result of the test	5 (0.84)	2 (0.34)	9 (1.5)
I do not have enough money for transportation	5 (0.84)	0	0
I think the examination is painful	4 (0.67)	0	1 (0.16)
I am afraid from getting breast cancer	4 (0.67)	0	5 (0.84)
I am not convinced that there is a benefit	3 (0.51)	5 (0.84)	5 (0.84)
I do not want to know even if I have breast cancer	2 (0.34)	2 (0.34)	5 (0.84)
I feel shy and embarrassed	1 (0.16)	3 (0.51)	10 (1.7)
I am afraid this could affect my family	1 (0.16)	0	0
I do not have enough money to pay for the test	1 (0.16)	0	5 (0.84)
I do not have health insurance	0	0	4 (0.67)
The nearby health centre has a male physician	0	0	7 (1.2)

*More than one answer per woman was possible.

## Discussion

This home visits intervention significantly improved women’s breast health knowledge, their perceived BSE knowledge, BSE practice and mammography screening. At first visit a low proportion of women reported practicing breast health examinations. Their previous mammography screening was significantly associated with being aged 40 years or older, having higher breast health knowledge and having attended a previous lecture about breast cancer. Women who received a free voucher and had a pre-set follow up visit were more likely to have mammography screening. Being busy and having other priorities was the most reported barrier for women’s breast health practices in the post-test, followed by not feeling at risk.

The participants in this study had relatively low breast health practices at the baseline. The reported practices in the pre-test indicated that previous mammography screening was associated with older age, higher breast health knowledge score and attending a previous lecture about breast cancer. This is consistent with Jordan’s breast health national guidelines that promote mammography screening starting from the age 40 years [7]. Public awareness can contribute to earlier detection of breast cancer if it is culturally appropriate and tailored to the specific population [11]. Educational programs that enhance women’s perceived self-efficacy and perceived benefits can lead to significant improvement in breast health practices [12]. Based on the Revised Health Belief Model, women’s perceived seriousness and susceptibility to illness influence their perceived threat, while, perceived benefits from early detection and perceived barriers influence health-seeking behaviour. In addition, general health motivation, perceived self-efficacy and ability enhance health-seeking behaviours [13]. Similarly, the social cognitive theory indicates that self-efficacy positively influences health behaviour and should be considered an integral component of educational interventions [14]. Therefore, breast health educational interventions that provide balanced health information might empower women to take an informed and more active decision-making role than they initially intended [15].

In this intervention, the post-test showed a significant improvement in women’s retained breast health knowledge, perceived BSE self-efficacy, reported BSE practice and mammography screening. Due to the increase in availability of and quantity of health promotion messages, women might be selective in the messages they receive and retain in their minds. Thus, health communication interventions should be more tailored and responsive to the targeted audience [16]. In a review of the specific challenges and proven interventions to improve attendance in female cancer screening in lower socioeconomic groups, it was found that consistently successful strategies were to offer free tests, eliminate geographical barriers, and to adopt an individually tailored pro-active communication addressing the specific barriers [17]. Thus, home visits by local outreach workers, as in our study, has been proposed to be more effective within this specific context in changing beliefs and practices than other breast health promotion strategies [17], as individual concerns and barriers can be addressed directly.

Our results demonstrated a higher use of the free mammography screening vouchers among the women who received the pre-set follow-up visit from the LCOW. This is consistent with the social support theory [18] as perceived support from the women’s social network, such as the LCOWs, is assumed to influence their health practices. Social support can be classified into formal or informal support [18]. Formal support is offered by health providers through giving information, guidance and advice. Informal support can be affective, appraisal, and instrumental and is considered the more effective type of support. Affective support involves mutual trust and genuine concern. Appraisal support confirms the self-value and is often provided by co-workers or through social influence. Instrumental support includes symbolic or material and tangible aid usually provided by family [18]. Mauad et al [19] found that home visits by outreach workers, who were well known in the community, were effective in improving the number of women screened for breast and cervical cancer in a low-income setting. The effectiveness of communication is enhanced since these outreach workers might share similar socio-cultural context with the women [19,20]. Our findings are also consistent with other studies, which have used local lay community workers to conduct educational sessions about breast cancer [21,22] and cervical cancer [23,24], which have shown significant increases in women’s screening participation rates.

Our study showed that receiving a voucher for free mammography screening increased the utilization of the screening facility. Out-of-pocket payments have been reported in literature as a barrier to screening [25]. Earp et al [21] described that an intervention including one-to-one conversations between lay community health advisors and women, use of culturally sensitive materials to promote breast cancer screening, increasing access to mammography by providing transportation and promoting lower charges, was more effective in increasing participation in mammography screening among lower income women than in the higher income group [21]. In a randomized trial by Stoner et al [26], vouchers for free mammography were distributed in two rural Minnesota counties and were significantly associated with increased participation in mammography screening [26]. In another intervention by Margolis et al [27], lay health advisors recruited women and offered screening appointments, resulting in a significant increased attendance in breast and cervical cancer screening [27].

In this study, being busy and having other priorities was the most reported barrier by women for their BSE, CBE and mammography screening practices. This is consistent with the findings of a qualitative study conducted by the same research team in Jordan [7], which indicated that Jordanian women were ambivalent to prioritize their own health and put children and family needs first. Lack of time and not feeling at risk have been reported also as common barriers for breast cancer screening among women in Asia [17,28].

This study has some limitations including recruitment of a non-random sample. Thus, our results cannot be directly generalized to other women in less privileged settings in Jordan. Although this study assessed the retained knowledge and changes in breast health practices six months after the home visits educational intervention, there is still a need to assess the long-term sustainability and the cost-effectiveness of such an intervention. Another limitation was that the LCOWs had only 625 free mammography screening vouchers to offer, which was not enough for all women who met the inclusion criteria. Additionally, a few follow-up visits could not be conducted. More importantly, we do not have complete follow-up data for more than half of all women who received a free voucher, which somewhat reduces the possibilities to identify the most relevant barriers for non-adherence to the referral for screening.

The strengths of this study are in the relatively large sample who received the first home visit, in the design that used multifaceted breast health promotion strategies, in the assessment of retained breast health knowledge and practices in a sub-sample six months after the first home visit, and the opportunity to track the actual utilization of the free mammography vouchers at the assigned mammography unit. Following mammography screening, women had all the necessary further investigations including: extra X-ray views, ultrasound, ultrasound guided biopsy and stereotactic biopsy. Two women who never had previous mammography screening before this home visits intervention used their free mammography screening vouchers and were diagnosed with stage II breast cancer. They were referred for treatment in the public sector.

## Conclusions

Evidence from this study suggests that home visits by trained LCOWs that incorporate tailored education about breast cancer and breast health, in addition to offering free mammography screening vouchers and a follow-up visit, were effective in improving women’s breast health knowledge and mammography screening in a less privileged area in Jordan. However, there is a need for further research to assess long-term sustainability and the cost-effectiveness of this type of intervention.

## Competing interests

The authors declare that they have no competing interests.

## Authors’ contributions

HT, RQ and RW conceived and designed the study to evaluate this home visits intervention, HT supervised data collection and data entry. HT, RQ, RW and LN conducted the data analysis. HT drafted the manuscript and VB, RQ, LN, HE and RW reviewed the manuscript. All the authors read and approved the final manuscript.

## Pre-publication history

The pre-publication history for this paper can be accessed here:

http://www.biomedcentral.com/1471-2458/14/428/prepub
